# Role of apoptosis repressor with caspase recruitment domain in human health and chronic diseases

**DOI:** 10.1080/07853890.2024.2409958

**Published:** 2024-10-01

**Authors:** Xiang Ao, Guoqiang Ji, Bingqiang Zhang, Wei Ding, Jianxun Wang, Ying Liu, Junqiang Xue

**Affiliations:** a Department of Rehabilitation Medicine, the Affiliated Hospital of Qingdao University, Qingdao, Shandong, P.R. China; b School of Basic Medicine, Qingdao University, Qingdao, Shandong, P.R. China; c Clinical Laboratory, Linqu People’s Hospital, Linqu, Shandong, P.R. China; d Institute for Restore Biotechnology, Qingdao Restore Biotechnology Co., Ltd, Qingdao, Shandong, P.R. China; e Key Laboratory of Cancer and Immune Cells of Qingdao, Qingdao Restore Biotechnology Co., Ltd, Qingdao, P.R. China; f Department of Comprehensive Internal Medicine, the Affiliated Hospital of Qingdao University, Qingdao, Shandong, P.R. China; g Institute for Translational Medicine, The Affiliated Hospital of Qingdao University, Qingdao Medical College, Qingdao University, Qingdao, Shandong, P.R. China

**Keywords:** ARC, apoptosis, chronic diseases, therapeutic target

## Abstract

Apoptosis repressor with caspase recruitment domain (ARC) is a highly potent and multifunctional suppressor of various types of programmed cell death (PCD) (e.g. apoptosis, necroptosis, and pyroptosis) and plays a key role in determining cell fate. Under physiological conditions, ARC is predominantly expressed in terminally differentiated cells, such as cardiomyocytes and skeletal muscle cells. Its expression and activity are tightly controlled by a complicated system consisting of transcription factor (TF), non-coding RNA (ncRNA), and post-translational modification (PTM). ARC dysregulation has been shown to be closely associated with many chronic diseases, including cardiovascular disease, cancer, diabetes, and neurodegenerative disease. However, the detailed mechanisms of ARC involved in the progression of these diseases remain unclear to a large extent. In this review, we mainly focus on the regulatory mechanisms of ARC expression and activity and its role in PCD. We also discuss the underlying mechanisms of ARC in health and disease and highlight the potential implications of ARC in the clinical treatment of patients with chronic diseases. This information may assist in developing ARC-based therapeutic strategies for patients with chronic diseases and expand researchers’ understanding of ARC.

## Introduction

Chronic diseases are the leading cause of death, accounting for approximately 70% of all deaths worldwide [1]. According to statistical data from the Centers for Disease Control and Prevention, six in ten adults have a chronic disease, and about four out of every ten have multiple chronic diseases [2]. A chronic disease refers to a diverse group of long-lasting diseases that occur for at least one or more years and require ongoing medical attention and/or restrict everyday life or certain activities [3]. Common chronic diseases include cardiovascular disease (CVD), cancer, diabetes, neurodegenerative disease, and liver disease [4–8]. Due to the aging population, urbanization, and lifestyle changes, cases of chronic diseases are rapidly increasing, and they have become a major public health problem worldwide [9], leading to patients and their families, and the whole society, being in intense pain and under huge economic burdens. The pathogenesis of chronic diseases is extremely complicated and still largely unknown, resulting in poor clinical outcomes for patients. Thus, clarifying the underlying mechanisms involved in the progression of chronic diseases may bring great benefits to the prevention and treatment of chronic diseases.

PCD pathways, such as apoptosis, necroptosis, and pyroptosis, are key players in various cellular processes that play vital roles in regulating cell turnover, tissue homeostasis, immune response, and other biological processes [10]. The dysregulation of PCDs is closely associated with the initiation and progression of multiple chronic diseases [11]. ARC, also known as nucleolar protein 3 (NOL3), is a highly potent and multifunctional regulator of PCDs that is widely expressed in various tissues and organs, including skeletal muscle, vascular smooth muscle, islet β cells, cochlear hair cells, primary spermatocytes, granulocytes, the brain, and the heart [12, 13]. It was first identified as an inhibitor of apoptosis that selectively targeted caspases in 1998 [14]. A previous study showed that ARC can suppress both extrinsic and intrinsic apoptotic pathways by engaging in nonhomotypic death-fold interactions [15]. Apart from apoptosis, ARC also plays a regulatory role in other PCDs, including necroptosis and pyroptosis [16, 17]. ARC has been shown to be involved in tissue regeneration and the modulation of numerous cellular functions [18–22]. Its expression and activity are tightly controlled by distinct mechanisms, such as ncRNAs [23], PTMs [24], and signaling pathways [25]. Evidence suggests that ARC dysregulation contributes to the progression of various chronic diseases, such as CVD, cancer, diabetes, and neurological diseases [13, 26, 27]. In this review, we mainly summarize recent findings regarding the structure, functions, and regulatory mechanisms of ARC and focus on its role in PCDs. We also explore the underlying mechanisms of ARC that are involved in health and chronic diseases and highlight its clinical implications for the treatment of patients with chronic diseases.

## Structure and functions of ARC

### Structural characteristics of ARC

**Figure 1.**
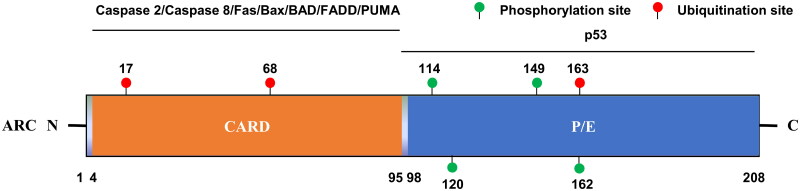
Structural characteristics and PTM sites of human ARC. ARC-interacting proteins are shown above the lines at the corresponding domains. ARC, apoptosis repressor with caspase recruitment domain; CARD, caspase recruitment domain; P/E, proline/glutamic; PTM, post-translational modification; FADD, Fas-associated protein with death domain; PUMA, p53-upregulated modulator of apoptosis.

The *NOL3* gene is located in the human chromosomal band 16q22.1, and it encodes an estimated 22.6 kDa multifunctional ARC protein consisting of 208 amino acids. The structure of ARC is evolutionarily conserved across species and mainly contains two domains – the N-terminal caspase recruitment domain (CARD) and a C-terminal region rich in proline/glutamic acid residues (P/E-rich region) (Figure 1). CARD (amino acids 4 to 95) exhibits significant homology to the pro-domains of the apical caspases as well as to the CARDs in Apaf-1 and RAIDD, and it mainly mediates the inhibitory effect of ARC on both extrinsic and intrinsic apoptotic pathways [12]. CARD is also responsible for the interaction of ARC with other proteins, including caspase 2, caspase 8, Fas, Fas-associated protein with death domain (FADD), p53-upregulated modulator of apoptosis (PUMA), and BCL2-associated X apoptosis regulator (Bax) [14, 15, 22, 28]. Furthermore, a crystal structure analysis revealed that the dimer structure of CARD consists of a five-helix bundle, which is crucial for its anti-apoptotic function [29]. The C-terminal P/E-rich region (nearly 110 amino acids) of ARC is highly acidic and can bind to considerable amounts of calcium, resulting in the inhibition of its interaction with procaspase 8 [30]. In addition, the P/E-rich region was found to mediate the interaction of ARC with tumor suppressor p53, thereby suppressing apoptosis [31].

### Functions of ARC

ARC is a well-studied apoptosis inhibitor that is required for development and the maintenance of homeostasis. It can play a protective role against cellular stress. For instance, Ekhterael et al. discovered that ARC blocked cytochrome c release by acting upstream of caspase activation, thereby protecting rat embryonic heart-derived H9c2 cells from apoptosis induced by hypoxia [32]. Zaiman et al. revealed that ARC protected pulmonary arterial smooth muscle cells from hypoxia-induced apoptotic death and promoted growth factor-induced proliferation and hypertrophy [33]. Wu et al. showed that ARC overexpression protected chick embryo cardiomyocytes from oxidative stress apoptosis and facilitated their survival [34]. ARC can exert its physiologic function by targeting other PCDs. For instance, in our previous study, ARC inhibited hydrogen peroxide-induced necrotic cardiomyocyte death by blocking the opening of the mitochondrial permeability transition (mPT) pore by targeting CypD. Consistent with this, ARC improved myocardial necrosis and long-term heart function in mice during ischemia/reperfusion (I/R) injury [16]. Furthermore, ARC was found to mediate the anti-pyroptotic actions of melatonin in cardiomyocytes exposed to oxygen–glucose deprivation/reperfusion (OGD/R) [17]. ARC is also involved in the regulation of tissue regeneration. Hu et al. demonstrated that ARC facilitated bone regeneration of bone marrow-derived mesenchymal stem cells by activating the Fgf-2/PI3K/AKT pathway [18]. In addition, ARC plays a crucial cell-autonomous role during neurogenesis. Kronenberg et al. discovered that both the number of doublecortin-positive cells and the number of calretinin-positive immature postmitotic neurons decreased in the hippocampus of ARC^-/-^ mice, indicating that ARC prevents cell death during adult granule cell neogenesis [35]. These findings strongly suggest that ARC plays a vital role in a variety of physiological and pathological processes, and that its dysregulation may contribute to the progression of various diseases.

## Molecular mechanisms of ARC regulation

**Figure 2.**
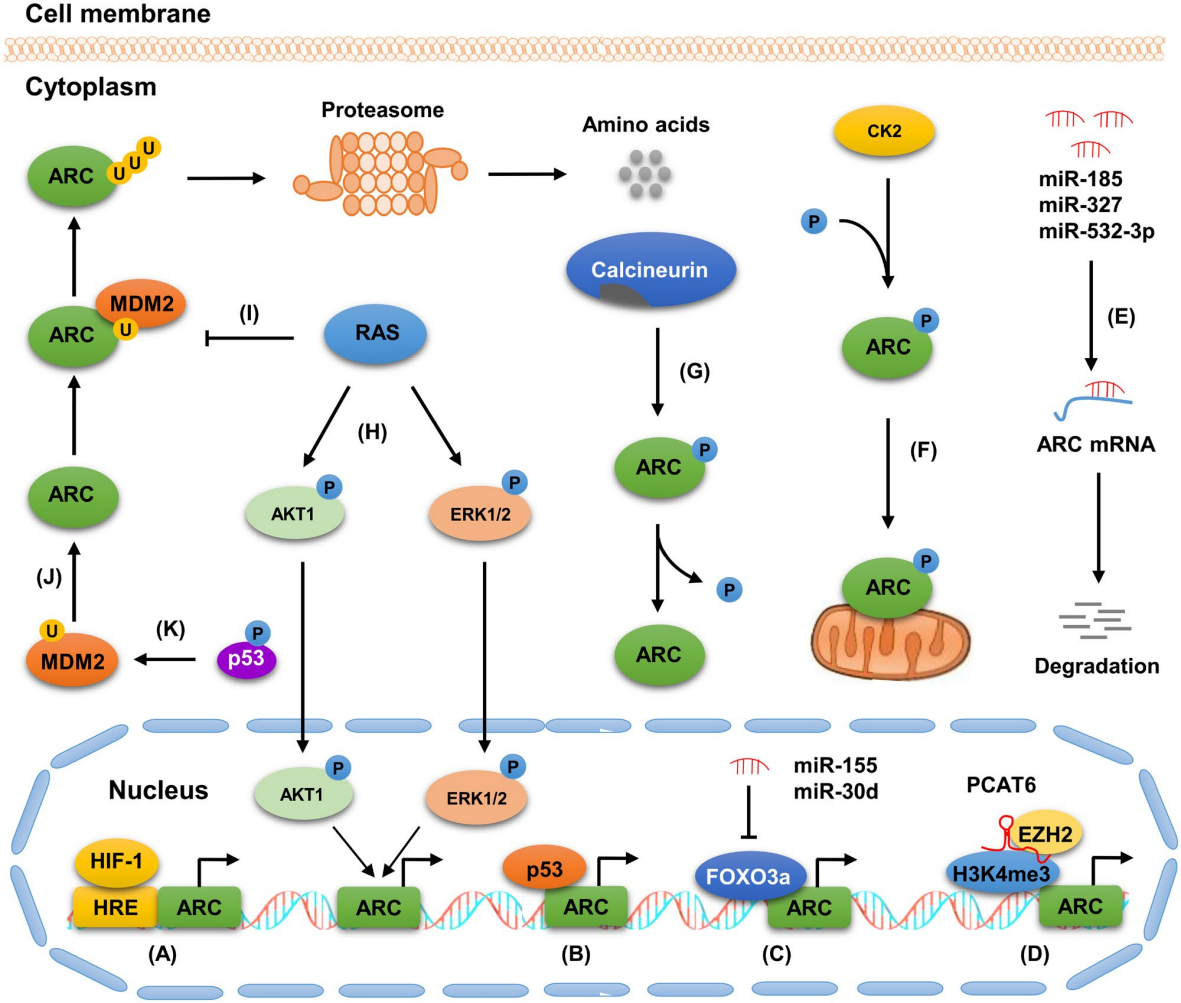
Regulation of ARC. The levels and cellular activity of ARC are modulated by various mechanisms, consisting of TFs, ncRNAs, and PTMs. (**A-B**), HIF-1 and p53, regulate ARC expression at the transcription level. (**C**), MiR-155 and miR-30d downregulates ARC by targeting FOXO3a. (**D**), lncRNA PCAT6 downregulates FADD by sponging miR-675. LncRNA PCAT6 upregulates ARC by recruiting EZH2 and H3K4me3 to its genomic region. (**E**), MiR-185, miR-327, and miR-532-3p facilitates ARC mRNA degradation by directly targeting it. (**F**), CK2 promote the nuclear translocalization of ARC by phosphorylating it at threonine 149. (**G**), Calcineurin mediates the dephosphorylation of ARC. (**H**), Ras facilitates ARC transcription by activating the AKT and ERK signaling pathways. (**I**), Ras inhibits ARC degradation by repressing its polyubiquitination. (**J**), MDM2 promotes ARC degradation by acting as its ubiquitin E3 ligase. (**K**), phosphorylated p53 facilitated the ubiquitin/proteasome-dependent degradation of ARC by upregulating MDM2.

The levels and cellular activity of ARC are tightly controlled by various mechanisms in different layers, including transcription, posttranscriptional, and post-translational levels (Figure 2). Herein, we summarize the main patterns of ARC modulation under physiological and pathological conditions.

### ARC regulation at the transcriptional level

ARC is a downstream target gene of several TFs, including forkhead box class O3a (FOXO3a), p53, and hypoxia-inducible factor 1 (HIF-1). Lu et al. showed that FOXO3a facilitated the expression of ARC by directly binding to its promoter, thereby maintaining cytoplasmic and mitochondrial calcium homeostasis and inhibiting cardiomyocyte apoptosis in the heart [36]. Consistent with this, FOXO3a knockdown was found to suppress ARC upregulation in H9c2 cells treated with melatonin [17]. Furthermore, Li et al. demonstrated that p53 knockdown in H9c2 cells significantly reduced ARC expression at both the protein and mRNA levels. Moreover, bioinformatics analysis revealed that the ARC promoter included an optimal p53 binding site, which was further validated by chromatin immunoprecipitation and luciferase reporter analysis [37]. These data strongly suggest that p53 can negatively modulate ARC expression at the transcriptional level. In addition, Ao et al. discovered that HIF-1α knockdown blocked hypoxia-induced upregulation of ARC. HIF-1α facilitated ARC expression by directly binding to hypoxia-responsive elements in the *NOL3* promoter [38]. Apart from TFs, oncogenic signaling pathways, such as Ras/mitogen-activated protein kinase (MAPK) and phosphatidylinositol 3-kinase (PI3K)/AKT signaling pathways, can also regulate ARC expression at the transcriptional level. For example, Wu et al. showed that the overexpression of activated N-Ras or H-Ras markedly upregulated ARC expression in MCF-10A normal and MDA-MB-231 breast cancer (BC) cells, whereas Ras knockdown in MCF-7 breast and HCT116 colon cancer (CC) cells significantly downregulated ARC levels. Mechanistically, N-Ras or H-Ras overexpression promoted the phosphorylation of AKT1 and ERK1/2, resulting in the transcriptional activation of the *NOL3* promoter and the stimulation of ARC mRNA production [39]. Consistent with this, the mRNA and protein levels of ARC significantly decreased in OCI-AML3 cells treated with PD0325901 (MEK/ERK inhibitor) or LY294002 (PI3K inhibitor) [25].

### Post-transcriptional regulation of ARC by ncRNAs

NcRNAs refer to unique RNA molecules with no or limited protein-coding capacity that participate in the modulation of almost all physiological processes [40]. Based on different characteristics, ncRNAs are classified into multiple categories, including microRNA (miRNA), long ncRNA (lncRNA), and circular RNA [41–43]. MiRNAs are well-studied regulatory ncRNA transcripts (18–25 nucleotides) that modulate the expression of specific genes by directly targeting their mRNAs [44, 45]. ARC has been shown to be a direct target of some miRNAs. In our previous work, we showed that miR-532-3p overexpression in cardiomyocytes resulted in a significant decrease in ARC expression, whereas its downregulation attenuated the reduction in ARC levels in cardiomyocytes treated with doxorubicin (DOX). A functional analysis revealed that miR-532-3p significantly downregulated ARC expression by directly targeting the ARC 3′-untranslated region (3′UTR), thereby enhancing the sensitivity of cardiomyocytes to DOX-induced mitochondrial fission and apoptosis [46]. In another study, we demonstrated that miR-185 decreased the resistance of gastric cancer (GC) cells to chemotherapeutic drugs by downregulating ARC *via* direct binding to its 3′UTR [47]. Furthermore, Li et al. discovered that miR-327 increased the levels of several pro-apoptotic proteins and the release of cytochrome c by targeting ARC at the post-transcriptional level, resulting in the facilitation of cardiomyocyte apoptosis and, ultimately, the promotion of I/R-induced myocardial injury [23]. In addition, ARC expression was found to be indirectly regulated by miR-155 and miR-30d. The two miRNAs decreased the levels of ARC by directly targeting FOXO3a [17, 48]. LncRNAs are vital modulators of ARC. For example, Huang et al. showed that lncRNA PCAT6 increased ARC levels by enhancing ARC transcriptional activity by promoting the recruitment of EZH2 and H3K4me3 to its genomic region, thereby facilitating cell growth and suppressing cell apoptosis in CC [49]. Collectively, these findings suggest that ARC expression is tightly modulated by an ncRNA-based network. ARC dysfunction induced by ncRNA dysregulation is closely associated with multiple chronic diseases, such as CVD and cancer. A complete understanding of the ncRNA-based regulatory mechanisms of ARC may provide new insights into the development of ARC-based individualized treatment of patients with chronic diseases.

### Contribution of PTMs to ARC regulation

PTMs (e.g. phosphorylation and ubiquitination) are reversible chemical modifications that are widely involved in the modulation of protein function and activity [50]. Growing evidence suggests that ARC can be modified by PTMs, including phosphorylation and ubiquitination. Li et al. showed that ARC was phosphorylated by casein kinase II (CK2) at threonine 149. The phosphorylation of this site mediated the anti-apoptosis function of ARC by facilitating its translocation to mitochondria and interaction with caspase 8 in cardiomyocytes [51]. Our previous work demonstrated that CK2-mediated phosphorylation of ARC can repress DOX-induced apoptosis and mitochondrial fission in cancer cells, resulting in the enhancement of drug resistance [24]. Furthermore, we revealed that calcineurin mediates the dephosphorylation of ARC. Calcineurin reduced the phosphorylation levels of ARC in cardiomyocytes treated with isoproterenol or aldosterone, and its suppression attenuated the reduction in ARC phosphorylation levels [52]. In addition, the phosphorylation of ARC mediated the response of cells to multiple stresses [53–55].

Ubiquitination, a dynamic process whereby proteins are tagged with ubiquitin chains, results in proteasome-mediated protein degradation [56]. ARC is a substrate for ubiquitination. In a study by Nam et al. endogenous ARC was found to be downregulated in response to death stimuli in cell and myocardial I/R mouse models. Further analysis revealed that ARC was polyubiquitinated during apoptosis. The mutation of potential ubiquitination sites in ARC resulted in the enhancement of ARC stability [57]. In another study, the mouse Double Minute 2 protein (MDM2) was identified as an E3-ubiquitin ligase of ARC. MDM2 mediated the ubiquitination of ARC, thereby facilitating ARC protein turnover in a proteasomal-dependent manner. Consistent with this, proteasomal inhibitors restrict ARC degradation induced by MDM2 [58]. Furthermore, Wu et al. showed that Ras knockdown significantly promoted ARC polyubiquitination, whereas mutations of the three ubiquitination sites in ARC reduced the enhancement of its polyubiquitination mediated by Ras knockdown, indicating that Ras enhanced ARC stability by repressing its polyubiquitination and degradation [39]. Taken together, these studies indicate that ARC can be regulated by multiple PTMs. A better understanding of the mechanism of the PTM network involved in ARC modulation may provide novel insights into the development of ARC-based therapeutic strategies for patients with chronic diseases.

## Implications of ARC in chronic diseases

ARC plays vital roles in the progression of various chronic diseases. However, the detailed mechanisms are not fully understood. Herein, we summarize the recent advances regarding the function of ARC in chronic diseases (Table 1).

**Table 1.** Functional roles of ARC in chronic diseases.

Disease types		Key messages	References
CVD	Hypertension	ARC was dramatically downregulated in skeletal muscle, heart, and aorta in spontaneously hypertensive rats compared with normotensive controls.	[59]
		ARC levels were remarkably increased in the lumen-occluding lesions and remodeled vessels of patients with pulmonary hypertension. ARC-deficient mice displayed reduced vascular remodeling under hypoxia. Mechanistically, ARC protected pulmonary arterial smooth muscle cells against hypoxia-mediated death.	[33]
		ARC knockdown significantly facilitated the apoptosis of PASMCs treated with serum deprivation by increasing the levels of proapoptotic factors and ROS, and decreasing the MMP.	[60]
		ARC was significantly downregulated in soleus muscle of spontaneously hypertensive rats. Moreover, The mass of ARC was 32 kDa in spontaneously hypertensive rats, but 30 kDa in WKY rat muscle.	[61]
	Myocardial I/R injury	ARC was identified as a direct target of miR-327 in myocardial I/R rats. ARC mediated the pro-apoptotic effect of miR-327 on cardiomyocyte in I/R-induced myocardial injury.	[23]
		TAT-ARC inhibited the activation of Bax by interacting with it, thereby preventing cytochrome c release in H9c2 cells treated with hydrogen peroxide. TAT-ARC also restricted cytochrome c release after I/R.	[22]
		ARC knockdown remarkably restricted melatonin-induced pyroptosis in H9c2 cells under I/R condition, whereas ARC overexpression had an opposite effect.	[17]
		The phosphorylation of ARC was decreased in the heart of I/R rat without anesthetic preconditioning. Conversely, anesthetic preconditioning remarkably upregulated ARC phosphorylation levels, decreased cytochrome c release after I/R.	[62]
		ARC was required for the restriction of endogenous estrogen on I/R-induced cardiomyocyte apoptosis.	[63]
		ARC improved myocardial necrosis and decreased the myocardial infarct size during I/R injury by repressing necrosis of cardiomyocytes by blocking mPTP.	[16]
	Cardiac hypertrophy	The levels of nuclear ARC were significantly downregulated in hypertrophic myocardial tissue and H9C2 cells. ARC overexpression or ARC export inhibition reduced norepinephrine or angiotensin II-induced hypertrophy by maintaining PHB in cells.	[64]
		ARC upregulation dramatically repressed Endothelin-mediated cardiomyocyte hypertrophy by blocking ROS production in a phosphorylation-dependent manner.	[55]
		CK2-induced phosphorylation of ARC suppressed cardiomyocyte hypertrophy by inhibiting the activation of mPT.	[54]
	DOX cardiotoxicity	ARC suppressed DOX-induced apoptosis by preventing Bax translocation to the mitochondrium and subsequently blocking the mitochondrial apoptosis in cardiomyocytes.	[65]
		ARC was phosphorylated by CK2, and its phosphorylation enhanced chemotherapy resistance by repressing DOX-induced apoptosis in cardiomyocytes.	[24]
		ARC overexpression reduced DOX-induced apoptosis in cardiomyocytes by inhibiting mitochondrial fission.	[46]
	Diabetic cardiomyopathy	ARC knockdown induced pyroptosis by increasing caspase-1 levels in cardiomyocytes under hyperglycemic conditions.	[48]
	HF	SNX13-mediated degradation of ARC activated the apoptosis of cardiomyocytes, thereby contributing to the development of HF.	[66]
Cancer	CRC	HIF-1-mediated upregulation of ARC inhibited TRAIL-induced apoptosis in SW480 colon cancer cells under hypoxia condition.	[38]
		ARC repressed colitis-correlated tumorigenesis by activating the NF-κB pathway in T cells *via* inhibiting the ubiquitination of TRAF6.	[67]
		ARC was upregulated in CC cells by PCAT6 *via* forming a complex with EZH2, resulting in the suppression of apoptosis and facilitation of CC progression.	[49]
		ARC expression was closely associated with the expression of MSH2 and MSH6 in CC liver metastasis. High ARC expression is essential for the inhibition of apoptosis.	[68]
		ARC was highly expressed in most CC cell lines and almost all primary CC. Cytoplasmic ARC in well, moderately, and poorly differentiated CC was upregulated, whereas nuclear ARC was only upregulated in moderately differentiated tumors.	[69]
	RCC	ARC was remarkably downregulated in RCC, but it strongly located in nucleus.	[70]
		ARC was highly expressed in ccRCC tissues.	[71]
		Silencing ARC significantly promoted TRAIL-induced apoptosis by activating caspase 8 in RCC cells. ARC also suppressed apoptosis by damaging mitochondrial activation. Moreover, silencing ARC remarkably facilitated Topotecan- and ABT-263-induced apoptosis in RCC cells.	[72]
		ARC was highly expressed in nearly 65% of RCC tissues, but it not expressed in control renal tissues. Moreover, ARC deficiency inhibited proliferation and promoted apoptosis in RCC cells. In SCID mice, ARC deficiency lead to significant inhibition of RCC tumor growth.	[73]
		ARC knockdown significantly enhanced sunitinib resistance in RCC SN12K1 cells by upregulating IL-6 and VEGF.	[74]
	AML	ARC was variably expressed in AML patients. AML patients with low or medium ARC expression exhibited longer overall survival and longer remission duration than patients with high ARC expression. ARC was an ideal biomarker that predicted survival in AML (*p* = 0.00013). Moreover, ARC suppression facilitated apoptosis and enhanced the sensitivity of OCI-AML3 cells to cytosine arabinoside.	[75]
		ARC was increased in AML and MSCs by birinapant. Birinapant-mediated upregulation was dependent on MAP3K14. ARC was negatively correlated with BIRC2. Moreover, silencing ARC in MSCs enhanced the sensitivity of co-cultured AML cells to birinapant-induced apoptosis.	[76]
		The levels of ARC were increased in AML cells co-cultured with MSCs. ARC upregulation enhanced the sensitivity of AML cells to cytarabine and apoptotic inducers.	[25]
		ARC upregulated IL1β in AML cells and increased the levels of CCL2, CCL4, and CXCL12 in MSCs by activating the NFκB pathway, thereby promoting the migration and adhesion of leukemia cells to MSCs.	[77]
		ARC was upregulated by β-catenin in AML cells. Mice transplanted with ARC-knockdown AML cells exhibited remarkably decreased leukemia burden, improved survival, and reduced resistance to chemotherapeutics.	[78]
	GC	ARC was significantly upregulated in GC cells. Silencing ARC remarkably promoted the apoptosis of GC cells induced low dose of DOX. ARC inhibited DOX-mediated mitochondrial fission by blocking the accumulation of Drp1 in mitochondria *via* binding to PUMA, resulting in the resistance of GC cells to DOX.	[28]
		ARC was a direct target of miR-185 and could mediate the effect of miR-185 on drug resistance in GC cells.	[47]
		ARC was a target of miR-532-3p.	[79]
	BC	Silencing ARC reduced primary tumor burden. ARC knockdown also repressed the invasion of BC cells, reduced the number of circulating cancer cells and lung metastases. ARC overexpression had the opposite effects. In addition, endogenous ARC enhanced drug resistance in primary tumors and invading cells.	[80]
		ARC repressed p53 tetramerization, thereby exposing the NES of p53 and subsequently inducing its translocation to the cytoplasm in BC cells.	[31]
	Nasopharyngeal carcinoma	High levels of ARC were significantly associated with advanced local invasion in nasopharyngeal carcinoma. ARC knockdown enhanced the sensitivity of NPC 6-10B cells to X-radiation and cisplatin. ARC overexpression exhibited the opposite effect.	[81]
	Melanoma	ARC repressed thapsigargin or tunicamycin-induced apoptosis in Me1007 cells by suppressing the activity of caspase 8. Moreover, ARC mainly localized in mitochondria and cytoplasm of melanoma cells.	[82]
	Glioma	ARC was dramatically upregulated in primary human glioma. Silencing ARC decreased cell viability and increased apoptosis in U251MG cells by activating caspase3/8 and facilitating Bax accumulation.	[83]
DM		ARC suppressed β-cell apoptosis by reducing the ER stress response *via* inhibiting CHOP induction. Silencing ARC in isolated islets enhanced palmitate-mediated apoptosis.	[84]
		ARC inhibited amyloid-induced β-cell apoptosis by inactivating the JNK pathway *via* directly binding to it.	[85]
		The levels of plasma glucose in ARC deficient mice was higher than wild type mice after HFD feeding.	[86]
LF		TAT-ARC repressed hepatocellular apoptosis in TNF-mediated LF mice by downregulating TNF-α *via* suppressing the JNK signaling.	[87]
		TAT-ARC suppressed acetaminophen overdose-induced LF by inhibiting acetaminophen-induced hepatocellular necrosis through the JNK pathway	[88]
AD		ARC was expressed in the hippocampal astrocytes. Moreover, ARC was dramatically upregulated in astrocytes administrated with Aβ25-35.	[89]
Hearing loss		ARC was downregulated in cochlear HCs administrated with neomycin. ARC knockdown remarkably enhanced the sensitivity of HEI-OC-1 cells to neomycin toxicity by upregulating pro-apoptotic factors, increasing ROS levels, and decreasing MMP.	[90]
Renal I/R injury		ARC was downregulated in the reperfused kidneys. ARC improved renal I/R injury by activating AKT/mTOR signaling pathway.	[91]
Colorectal familial adenomatous polyposis adenomas		ARC was highly expressed in the nuclei and cytoplasm of most colorectal familial adenomatous polyposis adenomas. Cytoplasmic ARC levels were closely associated with Bcl-2 levels, whereas nuclear ARC levels were closely associated with the expression of Bcl-2, COX-2 p53 and β-catenin.	[92]

CVD, cardiovascular disease; I/R, ischemia/reperfusion; DOX, doxorubicin; HF, heart failure; CRC, colorectal cancer; RCC, renal cell carcinoma; AML, acute myeloid leukemia; GC, gastric cancer; BC, breast cancer; DM, diabetes mellitus; LF, liver failure; AD, alzheimer’s disease.

### Role of ARC in CVD

CVD is the leading cause of deadly diseases that threaten human health and life around the world [93]. ARC was found to maintain normal cardiac functions by protecting cardiomyocytes from ischemic/hypoxic cell death [94]. Moreover, ARC-deficient mice exhibited significantly accelerated cardiomyopathy under cardiovascular ischemia or pressure overload when compared with those under resting conditions [95]. These data suggest that ARC dysregulation is involved in the development of CVDs.

#### ARC and hypertension

Hypertension is a well-recognized risk factor for CVDs, such as coronary heart disease, heart failure (HF), and stroke, which affect more than 1 billion adults and the aging population worldwide [59]. ARC dysregulation has been shown to be closely correlated with hypertension [33, 60, 96]. Quadrilatero et al. discovered that ARC was highly expressed in rat hearts and aortas. Moreover, ARC was significantly downregulated in the heart (-80%; *p* < 0.001) and the aorta (-71%; *p* < 0.001) of spontaneously hypertensive rats compared with normotensive rats [60]. Zhang et al. revealed that ARC was significantly upregulated in pulmonary arterial smooth muscle cells (PASMCs) exposed to serum deprivation, and its knockdown remarkably facilitated cell apoptosis. Mechanistically, ARC knockdown increased the levels of reactive oxygen species (ROS) and reduced the mitochondrial membrane potential (MMP) in PASMCs following serum deprivation stimulation, thereby enhancing the apoptosis of PASMCs [33]. In addition, in a study by Zaiman et al. high levels of ARC were observed in both remodeled vessels and lumen-occluding lesion of patients with pulmonary hypertension. Consistent with this, ARC-deficient mice exhibited reduced vascular remodeling and increased apoptosis in response to chronic hypoxia. Further analysis revealed that ARC protected PASMCs from hypoxia-induced death and promoted the proliferation and hypertrophy induced by the growth factor [96].

#### ARC and myocardial I/R injury

Myocardial infarction (MI) is considered the most serious form of coronary heart disease. Clinically, reperfusion is the most effective method for MI treatment. However, the restoration of blood flow can lead to irreversible damage to the myocardium, which is termed myocardial I/R injury [13]. Common factors contributing to I/R injury include oxidative stress, calcium overload, and mitochondrial dysfunction [16, 97]. Understanding the regulatory mechanisms of myocardial I/R injury may provide new insights for developing novel therapeutic strategies. Growing evidence shows that ARC plays a protective role during myocardial I/R injury. For instance, in our previous work, ARC was found to decrease myocardial infarct size and improve the long-term heart function of mice during I/R injury. Mechanistically, ARC blocked the opening of the mPT pores by targeting CypD, resulting in the inhibition of cardiac necrosis [98]. In another study, Gustafsson et al. explored the effect of ARC on myocardial I/R injury using the fusion protein TNF-mediated LF using the fusion protein ARC/HIV-1 transduction domain (TAT-ARC). They showed that ARC was able to protect adult rat hearts against I/R injury. Functional analysis revealed that ARC maintained MMP and nuclear morphology in cardiomyocytes, thereby protecting them from death induced by oxidative stress [99]. Furthermore, Chatterjee et al. discovered that stable transfection of ARC in a rabbit I/R model efficiently protected the heart from late postischemic cardiomyopathy [95]. Consistent with this outcome, ARC-deficient mice under biomechanical and ischemic stress exhibited accelerated cardiomyopathy characterized by cardiac enlargement and myocardial fibrosis [53]. In addition, ARC was found to mediate the cardioprotective effects of hydrogen sulfide and spironolactone during myocardial I/R injury [100, 101].

#### ARC and cardiac hypertrophy

Cardiac hypertrophy is the physiological response of the heart to various pressure stresses. Chronic cardiac hypertrophy is a common pathological process in multiple CVDs that ultimately progresses to HF [102]. Recent studies have shown that ARC dysregulation contributes to the progression of cardiac hypertrophy [54, 55, 64]. Murtaza et al. showed that the phosphorylation levels of ARC were dramatically downregulated in the hearts of angiotensinogen transgenic mice and in cardiomyocytes administrated with angiotensin II. Consistent with this, the suppression of ARC phosphorylation enhanced the hypertrophy of cardiomyocytes. A functional analysis revealed that ARC repressed cardiomyocyte hypertrophy by inactivating mPT [54]. In another study, they further demonstrated that ARC overexpression remarkably suppressed endothelin-mediated cardiomyocyte hypertrophy in a phosphorylation-dependent manner. Mechanistically, phosphorylated ARC inhibited endothelin-induced cardiomyocyte hypertrophy by blunting the ROS attack [55]. In addition, Xie et al. discovered that the nuclear ARC levels in H9c2 cells were significantly downregulated, whereas the total ARC levels remained stable. Further analysis revealed that nuclear ARC significantly weakened angiotensin II-induced hypertrophy by directly binding to prohibitin [64].

#### ARC and other CVDs

ARC also participated in the progression of other CVDs, such as DOX cardiotoxicity, diabetic cardiomyopathy, and HF. An et al. showed that ARC overexpression significantly enhanced the resistance of cardiomyocytes to DOX-induced apoptosis, whereas its knockdown exhibited the opposite effect. Functional analysis revealed that ARC improved DOX-induced cardiotoxicity by blocking the translocation of Bax from the cytosol to the mitochondria in cardiomyocytes [65]. Our previous work demonstrated that ARC overexpression reduced mitochondrial fission and apoptosis in cardiomyocytes treated with DOX by preventing the accumulation of dynamin-related protein 1 in mitochondria. Consistent with this outcome, ARC transgenic mice exhibited decreased cardiotoxicity following DOX treatment [46]. Furthermore, Li et al. showed that ARC degradation mediated by SNX13 downregulation promoted apoptosis in cardiomyocytes, resulting in HF [66]. In addition, ARC was found to increase the levels of caspase 1 and facilitate pyroptosis in cardiomyocytes in diabetic cardiomyopathy [48].

Taken together, these findings strongly suggest that ARC acts as an efficient inhibitor of PCDs (e.g. apoptosis, necroptosis, and pyroptosis) to play a protective role in the progression of various CVDs, including hypertension, myocardial I/R injury, cardiac hypertrophy, DOX cardiotoxicity, diabetic cardiomyopathy, and HF. However, the underlying mechanisms are still not fully understood. In-depth studies of ARC action in CVD progression will be of great importance for developing ARC-based therapeutic strategies for CVD treatment.

### Role of ARC in cancer

Cancer is the second leading cause of mortality after CVD globally [103]. High expression of ARC has been observed in multiple types of cancer, including colorectal cancer (CRC) [68], BC [80], renal cell carcinoma (RCC) [74], GC [79], and acute myeloid leukemia (AML) [75], indicating that ARC plays a crucial role in cancer progression.

#### ARC and CRC

CRC is the third most frequently diagnosed malignancy and the second leading cause of cancer-related death worldwide [75]. An estimated number of more than 1.9 million new CRC cases and nearly 0.9 million deaths occurred in 2020 [104]. Therefore, a better understanding of the mechanism underlying CRC progression is urgently required. Recent studies suggest that ARC dysregulation is correlated with CRC progression. Mercier et al. showed that the cytoplasmic levels of ARC were gradually increased in well, moderately, and poorly differentiated CC tissues compared with control tissues, whereas the nuclear levels of ARC were upregulated only in moderately differentiated CC tissues, indicating its potential as a diagnostic biomarker in CC [69]. In another study, the expression of ARC in CRC liver metastasis was found to be closely associated with the expression of MSH2 and MSH6 (DNA mismatch repair proteins involved in drug resistance) but was not correlated with p53 expression [68]. Furthermore, ARC was upregulated in the p53-deficient CC cell line SW480 in response to hypoxia. Mechanistically, HIF-1 increased the levels of ARC by directly binding to the hypoxia-responsive element in its promoter. Consistent with this, HIF-1α knockdown significantly attenuated hypoxia-induced ARC expression [38]. In addition, ARC was found to play a protective role in colorectal carcinogenesis. A functional analysis revealed that ARC suppressed the ubiquitination of TRAF6 by directly interacting with it, thereby activating the NF-κB pathway in T cells and subsequently resulting in the suppression of colitis-associated carcinogenesis [67].

#### ARC and RCC

RCC is the most prevalent urinary system cancer in adults, accounting for nearly 90% of kidney cancer cases globally. Despite some advances in diagnosis and treatment, the prognosis of RCC patients remains poor [105]. Therefore, it is imperative to elucidate RCC pathogenesis and to develop novel, effective therapeutic methods. Multiple studies have shown that ARC is involved in RCC progression. Rajandram et al. demonstrated that the whole-cell protein expression of ARC was significantly downregulated in ACHN RCC cells administrated with radiation and interferon-α; however, it exhibited remarkable nuclear localization [70]. In another study, high mRNA levels of ARC were detected in clear cell RCC (ccRCC) tissues, and ARC was co-expressed with the conventional biomarker CA9 in ccRCC cell clusters, indicating its potential role as a biomarker for ccRCC [106]. Moreover, Toth et al. discovered that ARC was remarkably increased in the cytoplasm and nuclei of ccRCC cells. A functional analysis revealed that ARC silencing enhanced the intrinsic apoptosis of ccRCC cells induced by topotecan and ABT263 through the mitochondrial pathway. ARC also suppressed extrinsic apoptosis by activating caspase 8 in ccRCC cells treated with TRAIL [72]. Razorenova et al. showed that ARC was significantly upregulated in approximately 65% of RCC tissue samples. ARC silencing inhibited cell growth survival in RCC cells. Consistent with this outcome, ARC deficiency led to a dramatic suppression of tumor growth in a mouse RCC model [73]. In addition, a different result was reported by Gobe et al. in which ARC was found to be dramatically decreased in most ccRCC, with its knockdown enhancing sunitinib resistance in RCC cells through the restoration of angiogenesis [74]. The pleiotropic effects of ARC in ccRCC remain controversial and require further exploration.

#### ARC and AML

AML is an infrequent and serious type of cancer in the hematopoietic system that results from genetic variations that lead to tumoral alterations and unbridled proliferation [107]. However, the mechanisms underlying AML oncology remain unclear. Thus, it is necessary to clarify the detailed mechanisms involved in AML progression and identify biomarkers and therapeutic targets. Some studies have demonstrated that ARC is a key regulator in the development of drug resistance in AML. For example, ARC was found to be variably expressed in AML patients, and its suppression facilitated apoptosis and enhanced the sensitivity of OCI-AML3 cells to Ara-C [75]. In another study, ARC was dramatically increased in AML treated with birinapant, whereas MAP3K14 silencing reduced ARC upregulation induced by birinapant. Moreover, ARC silencing in mesenchymal stromal cells (MSCs) facilitated birinapant-induced apoptosis in co-cultured AML cells [76]. Additionally, Mak et al. showed that the ectopic expression of ARC protected AML cells from chemotherapeutic drugs and apoptotic inducers, whereas its silencing obtained the opposite effects [25]. Carter et al. discovered that ARC increased the levels of IL1β in AML cells by activating the NFκB pathway, thereby promoting chemokine generation in MSCs and ultimately resulting in the enhancement of leukemia cell chemoresistance. Consistent with this, IL1β suppression attenuated the resistance of the cells co-cultured with MSCs to chemotherapeutic drugs [77]. They also found that ARC increased the COX-2 levels and PGE2 generation in MSCs by upregulating IL1β. PGE2 further promoted β-catenin expression, thereby facilitating ARC expression and enhancing drug resistance in AML cells [78]. These data suggest that ARC mediates drug resistance in AML through the regulation of leukemia–microenvironment interactions.

#### ARC and GC

GC is the fifth most frequently diagnosed malignancy and the third leading cause of cancer-related death globally. Despite its decreasing morbidity, the five-year survival rate of GC remains low due to the unclear mechanisms underlying GC development and the lack of effective therapeutic strategies [108, 109]. ARC is involved in the regulation of GC progression. Our previous work demonstrated that ARC was highly expressed in GC SGC-7901 cells and mainly localized in the cytoplasm. Further analysis revealed that ARC suppressed the apoptosis of SGC-7901 cells by repressing DOX-mediated mitochondrial fission by blocking Drp1 accumulations in mitochondria [28]. In another study, ARC was identified as a direct target of miR-185, which can mediate the promotional effect of miR-185 on the sensitivity of GC cells to chemotherapeutic agents [47]. In addition, Chen et al. showed that ARC was significantly decreased by miR-532-3p in CD320-overexpressed GC cells. The downregulation of ARC impaired mitochondrial functions by inducing the loss of MMPs and excessive generation of ROS, resulting in the activation of the cell apoptosis pathway in CD320-overexpressed GC cells [79].

#### ARC and other cancers

ARC is also involved in the carcinogenesis and progression of other cancers, including BC, nasopharyngeal carcinoma (NPC), melanoma, and glioma. Ramirez et al. explored the effect of ARC on BC progression using a transgenic mouse model and discovered that ARC knockout restricted the invasion of cancer cells and decreased the number of circulating tumor cells and lung metastases. Consistent with this outcome, ARC overexpression enhanced invasion and lung metastasis in BC [80]. In another study, nuclear ARC was found to repress p53 tetramerization by directing binding to it in BC cells, resulting in the translocation of p53 to the cytoplasm. ARC silencing in BC cells led to the accumulation of p53 in the nucleus, thereby activating the expression of p53 target genes, indicating that nuclear ARC was a negative modulator of p53 in BC cells [31]. Furthermore, Wu et al. showed that high ARC levels were closely associated with advanced local invasion in NPC. ARC knockdown enhanced the sensitivity of NPC 6-10B cells to X-radiation and cisplatin by activating caspase 2 and caspase 8, whereas its overexpression exhibited the opposite effects [81]. Chen et al. demonstrated that ARC overexpression in melanoma Me1007 cells enhanced drug resistance and suppressed chemotherapeutic drug-induced apoptosis by repressing caspase 8 activation. Conversely, ARC silencing sensitized resistant melanoma Mel-RM cells to apoptosis [82]. In addition, ARC was found to be highly expressed in gliomas compared to healthy brain tissues. The ARC-knockout U251MG cells exhibited decreased cell viability and increased apoptosis. A functional analysis revealed that ARC knockdown in U251MG cells enhanced VM-26 sensitivity by activating caspase 3/8 and facilitating Bax accumulation [83].

Overall, these findings suggest that ARC is remarkably upregulated in distinct types of cancer and plays an anti-apoptotic role in cancer progression by modulating multiple gene pathways. However, the detailed mechanisms of ARC underlying cancer progression are still unclear and controversial. In-depth studies on this aspect are urgently needed.

### Role of ARC in diabetes mellitus (DM)

DM is a complicated metabolic disorder that affects millions of people worldwide [110]. It is estimated that nearly 4.2 million adults aged 20–79 years died of DM, accounting for 11.3% of total deaths [111]. As a serious, chronic, and non-communicable disease, DM has become a major global threat to human health. Therefore, timely prevention and treatment of DM and its complications are urgently needed.

Loss of functional β-cells caused by apoptosis is considered the main pathological basis of DM [112]. McKimpson et al. discovered that ARC was expressed in more than 73% of human insulin-producing β-cells. A functional analysis revealed that ARC repressed the palmitate-induced apoptosis of β-cells by reducing the ER stress response *via* inhibiting CHOP. Consistent with this, ARC silencing in islets isolated from type 2 DM mice enhanced palmitate-induced apoptosis [84]. Templin et al. demonstrated that ARC overexpression reduced amyloid-induced β-cell apoptosis and loss, whereas its knockdown facilitated the amyloid-induced apoptosis of β-cells. Mechanistically, ARC decreased the amyloid-induced JNK phosphorylation by directly binding to JNK in β-cells, thereby inactivating the JNK pathway and ultimately resulting in the inhibition of apoptosis in the β-cells [85]. In another study, ARC-deficient mice exhibited higher levels of plasma glucose than control mice after high fat diet (HFD) feeding. Moreover, a remarkably reduced β-cell area was observed in HFD-fed ARC-deficient mice, indicating that the loss of ARC might aggravate hyperglycemia by diminishing β-cell compensation under HFD conditions [86]. Furthermore, cytoplasmic ARC was found to protect MIN6 cells (a widely used β cell line) from apoptosis, whereas the translocation of ARC to the nucleus promoted β cell apoptosis [113]. In addition, ARC was also involved in miR-30d-mediated pyroptosis and miR-532- mediated apoptosis in the cardiomyocytes of diabetic hearts [27, 48].

Collectively, these studies suggest that ARC participates in the progression of DM and its complications by regulating PCDs, such as apoptosis and pyroptosis. However, the exact role of ARC in DM progression remains largely unknown. In-depth studies are needed to clarify the detailed mechanisms involved.

### Role of ARC in other chronic diseases

Apart from the above-mentioned diseases, ARC dysregulation is also closely correlated with the progression of other chronic diseases, such as liver failure (LF), Alzheimer’s disease (AD), and hearing loss [87–90]. For example, An et al. explored the effect of ARC on TNF-mediated LF using the fusion protein TAT-ARC and discovered that the fatal LF induced by Jo2, ConA, or GalN/LPS was completely abolished in a mouse model treated with TAT-ARC. Mechanistically, ARC decreased TNF-α expression by inhibiting JNK activation *via* direct binding to JNK1/2, thereby preventing hepatocellular apoptosis in TNF-mediated LF mice [87]. They also found that TAT-ARC repressed acetaminophen overdose-induced LF by suppressing acetaminophen-induced hepatocellular necrosis through the JNK pathway [88]. Furthermore, in a study by Lv et al. the expression of ARC was observed in hippocampal neurons and astrocytes. Interestingly, the levels of ARC in hippocampal neurons were decreased in a time-dependent manner, whereas its levels in astrocytes were remarkably increased in Aβ25-35-induced neurotoxicity [89]. These data indicate that ARC is differentially expressed in distinct cell types in the hippocampus and that its aberrant expression plays complicated roles in amyloid-related diseases, such as AD. In addition, Guan et al. showed that ARC was dramatically downregulated in cochlear hair cells (HC) and the cochlear hair cell-like cell line HEI-OC-1 treated with neomycin. ARC knockdown remarkably enhanced neomycin sensitivity in HEI-OC-1 cells by upregulating pro-apoptotic factors, decreasing MMP, and increasing ROS, suggesting that ARC might be involved in the progression of hearing loss by serving as a key regulator of HC apoptosis [90].

**Figure 3.**
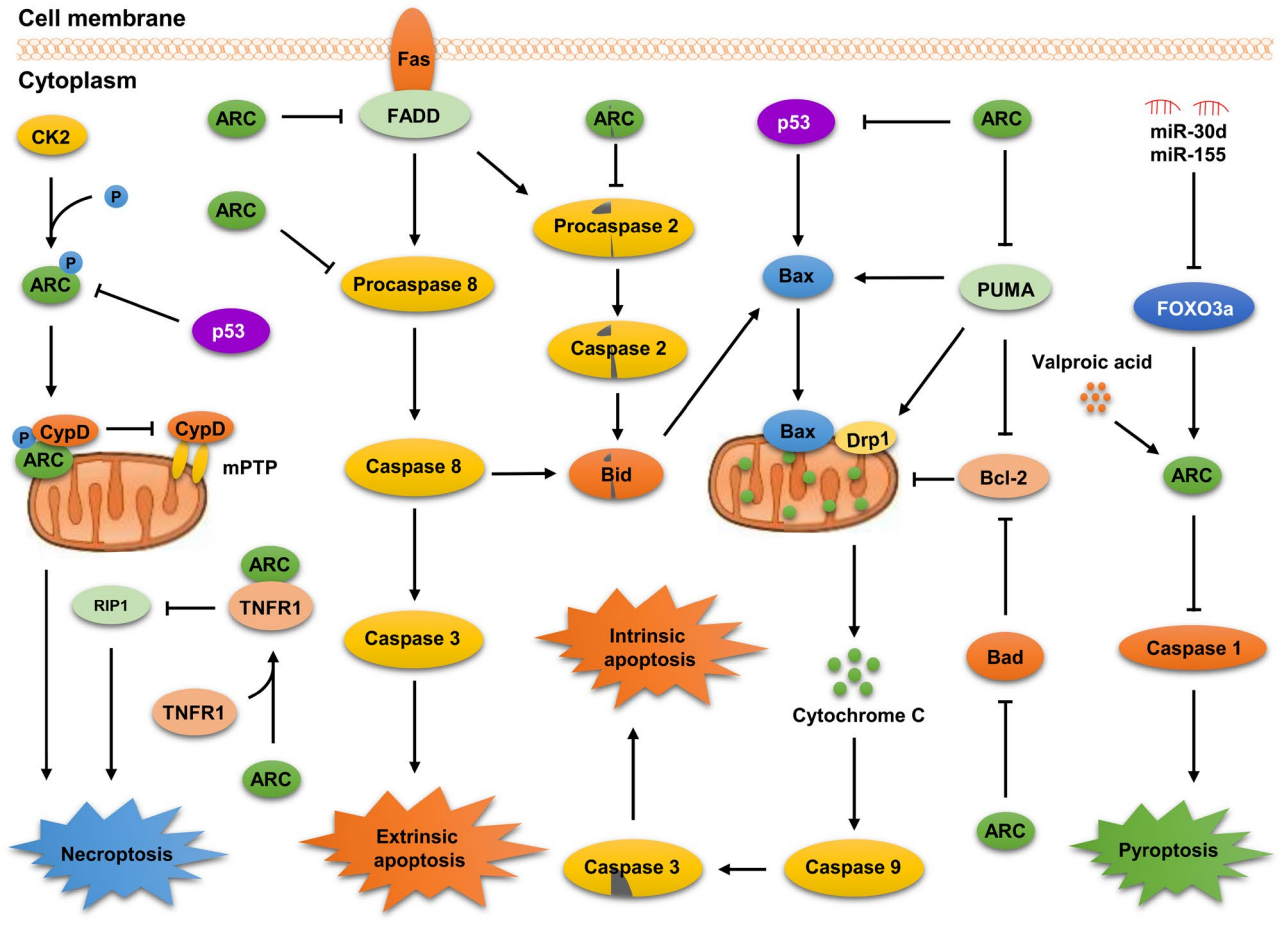
Mechanisms of ARC in regulating the PCDs. The roles of ARC in distinct PCDs (apoptosis, necroptosis, and pyroptosis) was simplified in a diagram. The diagram exhibits the key components and the reactions of PCDs.

In summary, ARC plays a pleiotropic role in a series of chronic diseases, including CVD, cancer, DM, LF, AD, and hearing loss. The underlying mechanisms of ARC involved in these diseases mainly depend on its modulation on various PCDs, such as apoptosis, necroptosis, and pyroptosis (Figure 3). A full understanding of its mechanisms in the progression of chronic diseases will be highly beneficial to the development of ARC-based therapeutics.

## Clinical applications of ARC in chronic disease

Due to its vital role in chronic diseases, ARC has exhibited great potential as a therapeutic target in clinics. ARC has been shown to play a protective role in multiple CVDs, including hypertension, MI, cardiomyocyte hypertrophy, DOX cardiotoxicity, diabetic cardiomyopathy, and HF [46, 48, 55, 60, 66, 98]. Low expression of ARC is observed in cardiomyocytes during CVD progression, and its downregulation contributes to the formation and development of these CVDs. Therefore, a targeted increase in ARC expression may represent a valuable strategy for improving CVD treatment. Moreover, ARC is upregulated in a series of cancer types, such as CRC, BC, RCC, GC, and AML [68, 74, 75, 80, 114]. ARC upregulation facilitates cancer progression by inhibiting PCDs and enhancing chemoresistance in tumor cells. Thus, silencing ARC may be an effective way to repress cancer progression and reverse chemoresistance. Given that ARC is a direct target of multiple ncRNAs, such as miR-532-3p, miR-327, and PCAT6 [23, 46, 49], the therapeutic modulation of ARC-targeting ncRNAs is recognized as a promising strategy for the treatment of chronic diseases. Furthermore, the identification or synthesis of new drugs targeting ARC is also a potential strategy for patients with chronic diseases. In addition, the unique, differentially expressed pattern endows ARC with great value as a biomarker for chronic diseases. For instance, ARC was found to be variably expressed in AML patients. AML patients with low or medium ARC expression exhibited longer overall survival (*p* = 0.0015) and longer remission duration (*p* = 0.000) compared with those with high ARC expression, indicating that ARC was an independent prognostic biomarker in AML treatment [75].

## Conclusion

Chronic diseases are the leading cause of global death, threatening patients’ lives to a great extent. The pathological mechanisms of chronic diseases are highly complicated and not yet fully clarified. Accordingly, a better understanding of the mechanisms involved in chronic diseases will be of great value in the development of efficient therapeutics. A number of studies have shown that ARC plays an essential role in various biological processes by serving as a multifunctional regulator of PCDs, including apoptosis, necroptosis, and pyroptosis. Its expression and functions are tightly modulated by a complex system consisting of ncRNAs, signaling pathways, and PTMs. Consequently, ARC dysregulation is remarkably correlated with the formation and progression of various chronic diseases, such as CVD, cancer, and neurological diseases [74, 94]. Moreover, ARC displays great potential as a therapeutic target or biomarker due to its key role and differentially expressed pattern in chronic diseases. Therefore, identifying or synthetizing novel drugs targeting ARC is a valuable therapeutic strategy for patients with chronic diseases. In addition, ARC has been identified as a target of multiple miRNAs and lncRNAs, indicating that these ncRNAs are also promising therapeutic targets for patients with chronic diseases.

## Limitations and future perspectives

Although ARC has exhibited some exciting value in the clinical treatment of chronic diseases, some unavoidable limitations still exist. For example, targeting ARC may result in unpredictable pathological reactions due to its essential role in normal physiological processes. Nevertheless, recent studies strongly suggest that ARC possesses great potential as a therapeutic target or biomarker for patients with chronic diseases. In-depth investigations of the underlying mechanisms of ARC in chronic diseases will be of great benefit to the development of ARC-based therapeutics in clinics.

## Data Availability

Data sharing is not applicable to this article as no new data were created or analyzed in this study.
